# The transcriptome of the newt *Cynops orientalis* provides new insights into evolution and function of sexual gene networks in sarcopterygians

**DOI:** 10.1038/s41598-020-62408-x

**Published:** 2020-03-25

**Authors:** Maria Assunta Biscotti, Federica Carducci, Marco Barucca, Marco Gerdol, Alberto Pallavicini, Manfred Schartl, Adriana Canapa, Mateus Contar Adolfi

**Affiliations:** 1 Dipartimento di Scienze della Vita e dell’Ambiente, Università Politecnica delle Marche, Ancona, Italy; 2 Dipartimento di Scienze della Vita, Università di Trieste, Trieste, Italy; 3 Stazione Zoologica Anton Dohrn, Napoli, Italy; 4 Developmental Biochemistry, Biocenter, University of Wuerzburg, Wuerzburg, Germany; 5 Comprehensive Cancer Center Mainfranken, University Clinic Wuerzburg, Wuerzburg, Germany; 6 Hagler Institute of Advanced Study and Department of Biology, Texas A&M University, and Xiphophorus Genetic Stock Center, Texas State University, San Marcos, USA

**Keywords:** Developmental biology, Evolution

## Abstract

Amphibians evolved in the Devonian period about 400 Mya and represent a transition step in tetrapod evolution. Among amphibians, high-throughput sequencing data are very limited for Caudata, due to their largest genome sizes among terrestrial vertebrates. In this paper we present the transcriptome from the fire bellied newt *Cynops orientalis*. Data here presented display a high level of completeness, comparable to the fully sequenced genomes available from other amphibians. Moreover, this work focused on genes involved in gametogenesis and sexual development. Surprisingly, the *gsdf* gene was identified for the first time in a tetrapod species, so far known only from bony fish and basal sarcopterygians. Our analysis failed to isolate *fgf24* and *foxl*3, supporting the possible loss of both genes in the common ancestor of Rhipidistians. In *Cynops*, the expression analysis of genes described to be sex-related in vertebrates singled out an expected functional role for some genes, while others displayed an unforeseen behavior, confirming the high variability of the sex-related pathway in vertebrates.

## Introduction

Amphibians represent a group of vertebrates containing over 7,100 species worldwide^1^. These organisms evolved in the Devonian period about 400 Mya and represent a transition step in tetrapod evolution^2^. Modern-day amphibians have diverged into three orders with distinct anatomical features: Gymnophiona (caecilians, limbless amphibians), Caudata (salamanders and tritons), and Anura (frogs and toads). Amphibians have many features that make them ideal as animal models because of their physiology, diversity, phylogeny, and amazing capability of tissue regeneration. On the other hand, amphibians represent a case of global biodiversity crisis since they are among the most threatened species in the world^3^.

The Chinese fire-bellied newt, *Cynops orientalis* (David, 1873), is a small (6–10.3 cm long) urodele (Salamandridae: Pleurodelinae) distributed in Asia. It shows a color ranging from dark brown to black on the back and with belly from orange to scarlet red with numerous black spots. The sexual dimorphism is evident in size (males are smaller than females) and shape of tail and cloaca. *C. orientalis* lives in ponds and marshes and prefers cold and calm waters with a bottom of mud. In nature, in the Chinese region of Changsha, the reproduction period spans from March to July, when water temperatures range from 15 to 23 °C, and juveniles reach sexual maturity from 1 to 3 years.

Data obtained by whole genome sequencing (WGS) are a very valuable resource to understand the evolutionary history and biology of species. Despite the importance of amphibians, the genomes of only 13 species have been fully sequenced as of January 2020. The genomes of newts contain a large proportion of repetitive sequence and are the largest ones among terrestrial vertebrates^4^. These factors currently represent a significant challenge for whole genome assembly^5^, in particular due to the high computational resources required for handling the large amount of input sequencing data and the technical limitations of most assembly algorithms, optimized for genomes with a size comparable with human or smaller. The only exception was the axolotl genome (~32 Gb)^6^, which has been produced through a huge joint effort and at high costs, together with the development of *ad hoc* bioinformatics tools and computational strategies to manage the unusual size of the assembly.

RNA sequencing technologies represent a cost-effective tool for large-scale transcriptome profiling that allows the rapid and complete elucidation of transcript sequence information in a specific tissue, developmental stage or physiological condition. Currently, most of published works in urodeles are focused on limb regeneration^7^ and very few from the Pleurodelinae^8–10^. Data on the expression and function of genes participating in sexual development and gametogenesis in urodeles and in general in amphibians are very limited^11–18^. The investigation of these processes in this taxonomic group is extremely interesting for the variety of mechanisms involved in sexual development present in Anura and Caudata and for their phylogenetic position. In general, the fate of the undifferentiated gonad towards male or female development is determined by genetic or environmental mechanisms. However, a great number of studies provides increasing evidence that the master sex determination gene and genes involved in the downstream sex differentiation network are extremely variable even between closely related species^19,20^. Thus, knowledge on newts can provide insights on the gene network acting in these organisms and its evolution. Indeed, salamanders belong to Caudata for which little information is available compared to the sister group Anura.

In this paper we report the assembly of a *Cynops orientalis* transcriptome, which is characterized by a high level of completeness and provides one of the most comprehensive molecular resources for amphibians. Moreover, transcriptome data obtained from testes and ovaries were exploited to investigate genes involved in gametogenesis and sexual development. We report for the first time the identification of the important sex determining *gsdf* gene in tetrapods, so far known only from bony fish, lungfish and coelacanth. In particular, our data represent a useful tool for future studies involving gene expression experiments, comparative transcriptomics, genomics, and metabolomics in amphibians.

## Results and Discussion

### Sequencing, *de novo* assembly, and annotation

Upon quality trimming, the total sequencing output was ~422.5 million paired-end reads obtained from male and female gonads, and female liver in three biological replicates (Supplementary Table S1). The non-redundant transcriptome assembly obtained from these three tissues comprised 45,831 contigs, whose size ranged from 201 to 22,390 base pairs, with a mean length of 1,594 bp and N50 length of 3,475 bp, accounting for a total assembly size slightly higher than 78 Mb.

**Figure 1:**
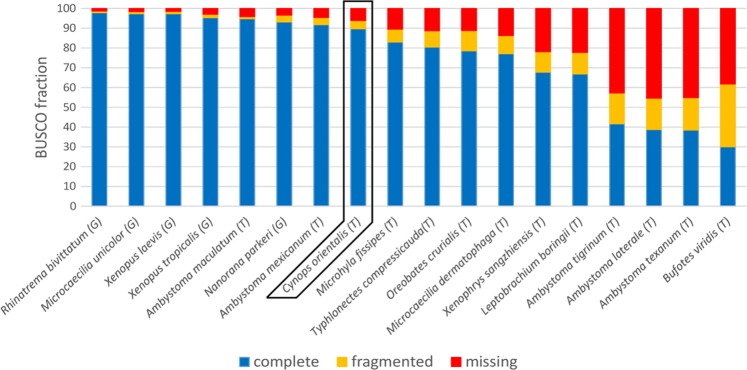
Comparative qualitative assessment of selected genomic and transcriptomic resources currently available for Amphibia, based on the evaluation of complete, fragmented and missing metazoan BUSCOs from OrthoDB v.9. G: genome; T: transcriptome.

The reference transcriptome displayed a high level of completeness, with just 6.2% tetrapod BUSCOs being absent. At the same time, it contained a very high fraction of contigs containing complete open reading frames, as revealed by the detection of 89.7% complete and just 4.1% fragmented tetrapod BUSCOs. Overall, the quality of the *C. orientalis* transcriptome, both in terms of number of orthologs present and in terms of complete BUSCOs identified, was significantly higher than most available amphibian reference transcriptomes^6,21–23^. Completeness and fragmentation rates were comparable with the outcome of the most recent high-depth approaches targeting multiple tissues^24–26^ (Fig. 1, Supplementary Table S2).

**Figure 2:**
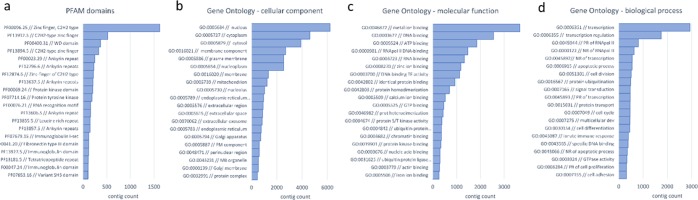
Top 20 most abundant PFAM domains (panel a), Gene Ontology cellular component terms (panel b), molecular function terms (panel c) and biological process terms (panel d) in the *C. orientalis* transcriptome.

A total of 19,632 contigs contained an ORF longer than 100 codons, and this number closely matched the estimates of the full complement of protein-coding genes of diploid tetrapods (e.g. 20,454 in human, 18,442 in *Xenopus tropicalis* and 18,595 in the *Anolis carolinensis*). Overall, 19,961 contigs obtained a positive BLAST hit, which resulted in the annotation of 41.61%, 38.76% and 31.20% sequences with the Gene Ontology, KEGG and eggNOG resources, respectively. The inspection of the most abundant Pfam domains and Gene Ontology terms (Fig. 2) confirmed the comparability of the assembled transcriptome with the complete repertoire of protein-coding genes encoded by tetrapod genomes. Indeed, the most represented annotations closely mirrored the distribution observed in other Amphibia, as exemplified by the *Xenopus tropicalis* genome^27^, with a widespread representation of domains with structural function (e.g. ankyrin repeats, WD40 repeats), marked protein-protein interaction properties (e.g. immunoglobulin and fibronectin type III domains) or ion-binding potential (e.g. C2H2 zinc finger motifs). In light of these observations, the *C. orientalis* transcript collection is one of the most complete available mRNA collections for amphibians and provides a reference for comparative genomics studies of comparable quality to the fully sequenced amphibian genomes of *Xenopus laevis*^28^ and *Nanorana parkeri*^29^, and just slightly lower than *Xenopus tropicalis*, *Microcaecilia unicolor* and *Rhinatrema bivittatum*^27^. Newts (Salamandridae, Pleurodelinae) have very large genomes even if compared with amphibian standards (usually > 20 Gb)^30^. In particular, the nuclear DNA content of *C. orientalis* has been previously estimated at 44.27 Gb, exceeding the size of axolotl (32 Gb), making the attainment of a complete genome assembly for this species unlikely in the foreseeable future due to the high sequencing costs and computational requirements that would be required to achieve this task.

The Chinese fire-bellied newt transcriptome, with its high level of completeness and high proportion of contigs containing complete open reading frames, most certainly meets the requirements that might potentially allow its use as a temporary genome substitute until further technological advancements will finally allow to obtain a complete reference genome sequence.

While a few other Pleurodelinae transcriptomic studies have been previously published or are currently in progress^8,9^, the resources available for *Cynops* spp. are still limited and no high-throughput data is available for *C. orientalis*. To date, the phylogenetically closest species, which has been the subject of RNA-sequencing is the congeneric Japanese fire-berry newt *Cynops pyrrhogaster*^10^. However, this study has been targeted on eyeballs, to study the retinal regeneration process, and therefore it does not provide the opportunity to investigate gene expression profiles from multiple tissues.

### General overview on gene expression profiles

**Figure 3:**
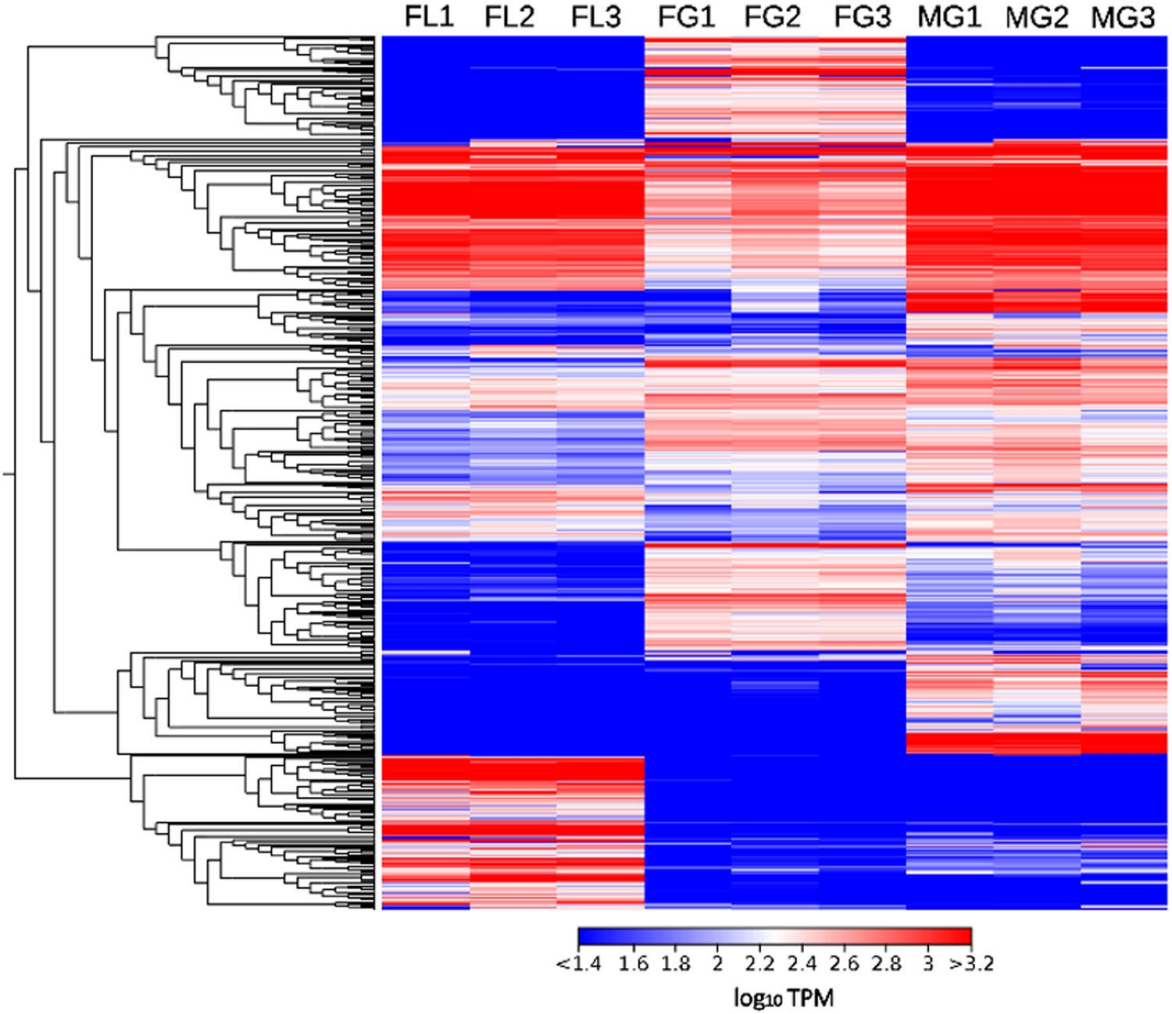
Heat map, providing an overview on gene expression profiles of the nine newt samples. Log_10_ TPM gene expression values were hierarchically clustered, based on an average method and correlation-based dissimilarity matrix. Only transcripts achieving TPM values > = 200 in at least in one sample are shown.

The *C. orientalis* liver, male and female gonads displayed highly distinct gene expression profiles (Fig. 3), which is consistent with the highly specialized function that these organs carry out in newt biology and with observations previously collected in other tetrapods^31,32^. The three biological replicates did not display any significant variation in pairwise comparisons, as revealed by both heat map (Fig. 3) and PCA graphical representations (Supplementary Fig. S1).

As expected, the liver, a tissue extensively involved in biosynthetic processes, displayed the lowest diversity of expressed mRNAs. The three samples were characterized by a low Transcriptomic Diversity Index (TDI), between 1.98 and 2.17^33^, with about 50% of the global transcriptional effort directed towards the production of ~100 highly expressed mRNAs. On the other hand, a broader range of transcripts were expressed in male and female gonads, with TDIs in the range of 2.76–3.24 and 3.45–4.39, respectively (Supplementary Fig. S2). These values to some extent explain the very high completeness of the transcriptome assembly, in spite of its construction from just three adult tissues. The rich transcriptomic landscape of testis could be related to the increased accessibility of chromatin to transcription factors in meiotic and early haploid spermatogenic cells^34^, which results in promiscuous transcription^35–37^.

Compared with testis, female gonads displayed an even richer transcriptional pattern. This observation might be more intimately linked to the function of amphibian oocytes and with the extensive presence of lampbrush chromosomes in these cells^38^. Widespread transcription of a broad range of mRNA types, which appear to be up-regulated compared with somatic cells, has been previously reported in many amphibian species. In *Xenopus*, this high transcriptional activity has been hypothesized to play an important role in preparation for the rapid differentiation of cell lines in early embryonic stages^39^.

### Analysis of gonad transcriptomes

**Figure 4:**
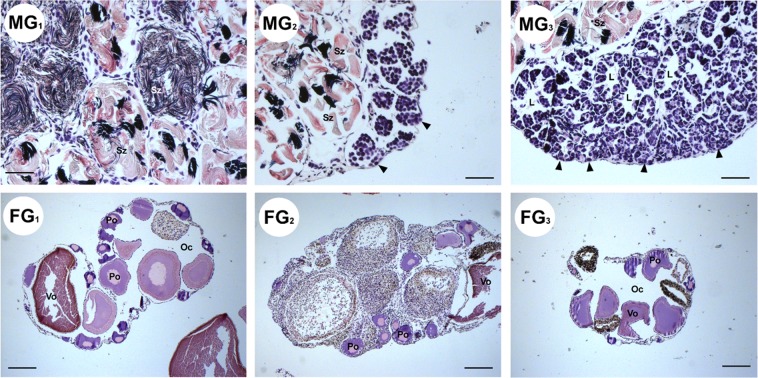
Morphology of *C. orientalis* gonads. Testes of all males (MG1, MG2 and MG3) are mature containing high numbers of spermatozoa. The spermatogonia are located in a more cephalic position and confined to cysts. Scale bars = 100 µm. All female gonads (FG1, FG2 and FG3) are mature presenting germ cells in advanced oogenesis stages. Lumen (L), ovarian cavity (Oc), pre-vitellogenic (Po) oocyte, spermatogonia (arrow), spermatozoa (Sz), and vitellogenic oocyte (Vo). Scale bar = 400 µm.

Histological analysis of the gonads confirmed the sex and the gonadal maturation stage of the animals (Fig. 4). Testes had the typical lobular structure of salamanders, and each lobe contained several cysts with spermatogenic cells in different stages of development. The gonads of all male individuals presented a high number of spermatozoa, the cysts displaying a cephalo-caudal gradient with respect to the stage of spermatogenesis^40^. The ovaries of all females contained oocytes at different stages of oogenesis, including vitellogenic and highly pigmented oocytes^41^.

The most highly expressed genes in female gonads were histones, which cumulatively accounted for ~15% of the transcriptome of this tissue and displayed expression levels >1,000 folds higher than testis (Supplementary Table S3). The high expression levels of many histone types (e.g. H1, H2A, H2B, H3 and H4) is in line with the significant stage-dependent histone modifications occurring during oocyte development in many tetrapods, including amphibians^42^ and mammals^43^. Some of the histone isoforms identified closely matched the *Xenopus* H2AX variants, which have been previously shown to be highly enriched in oocytes and early embryos^44^, the human oocyte-specific histone H1^45^, and accessory histone linker proteins such as the *Xenopus* B4 protein^46^. Due to the accepted role of oocyte-specific histones in maintaining genome stability in the female germ line^42,43^, these nuclear proteins might hold a particularly important role in organisms with a genome whose size exceeds by >10 folds the average size of mammals.

Other genes abundantly expressed in female gonads included several molecular players involved in the regulation of meiosis and cell cycle (e.g. cyclins and geminin^47^, as well as in DNA synthesis (e.g. the ribonucleotide reductases), oocyte maturation and early embryonic development. With this regard, the strong expression of perilipins (>1,500 TPM) and glycogenin-1 (>1,000 TPM) is consistent with the important role of these proteins in the formation of oocyte lipid droplets^48,49^ and glycogen storage^50^, respectively. The strong female specificity of Cyclin A1 (expression level >1,000 TPM, nearly 3,000 folds higher than testis) is noteworthy, since it displayed a strikingly opposite trend compared to mammals. In human testis cyclin A1 is a master regulator of gametogenesis; and it is only expressed in female gonads in cases of ovarian cancer^51^.

Male gonads displayed a generalized lack of dominant tissue-specific transcripts, exception made for protamines. However, these were probably not identified due to their extreme low complexity. The majority of the most highly expressed genes were not directly linked with gametogenesis, being rather involved in housekeeping functions (e.g. ribosomal and cytoskeletal proteins), which were usually found expressed at levels 5–10 times higher than ovary (Supplementary Table S4). There were, however, some key exceptions, such as high expression of ornithine decarboxylase antizyme 3, which is linked with polyamine biosynthesis, a fundamental process to ensure sperm motility^52^. The dual specificity phosphatase DUSP13, which shows skeletal muscle and testis-specificity in human (based on GTEx data, https://www.gtexportal.org/home/) also displayed an expression level >1,000 folds higher in testis than in ovary. Similarly, the tubulin polymerization promoting protein TPPP2, which is expressed in a testis-specific manner in humans, showed this feature also in the newt. The biological significance of other “out of place” transcripts, expressed at much higher levels in testis compared with ovary remains to be fully elucidated.

### Genes potentially involved in gonad sex differentiation and gametogenesis in *Cynops orientalis*: expression analysis and evolutionary considerations

The set of genes involved in sex differentiation and gametogenesis analyzed previously in basal sarcopterygians^53^ was investigated also in *C. orientalis* to increase knowledge in early tetrapods. This analysis performed on gonad transcriptomes allowed to retrieve 38 transcripts annotated from genes involved in sexual development (Table 1), which show complete CDSs except for 6 transcripts (Supplementary Table S5).

**Table 1:** Genes involved in sex differentiation and gametogenesis studied in *Cynops orientalis*.

Growth factor	Transcription factors	Meiosis regulation genes	Steroidogenic enzymes	Steroidogenic receptor genes	Follistatin and β catenin
*amh*	*dmrt1*	*aldh1a1*	*sdr5a1*	*ar*	*fst*
*amhr2*	*dmrt3*	*aldh1a2*	*sdr5a2*	*esra*	*ctnnb1*
*cxcl12*	*dmrt6*	*aldh1a3*	*sdr5a3*	*esrb*	
*cxcr4*	*foxl2*	*cyp26a1*	*cyp11b*		
*cxcr7*	*foxl3*	*cyp26b1*	*cyp19a1*		
*dhh1*	*lrppc*	*cyp26c1*			
*fgf9*	*dax1*	*stra8*			
*fgf16*	*pax2a*				
*fgf20*	*sf1*				
*fgf24*	*sox3*				
*gsdf*	*sox5*				
*pdgfa*	*sox8*				
*pdgfb*	*sox9*				
*pdgfra*	*sox10*				
*pdgfrb*	*wt1*				
*rspo1*	*gata4*				
*wnt4*					
*ptch2*					
*hhip*					

Underlined genes were not found in *Cynops orientalis* transcriptomes.

**Figure 5:**
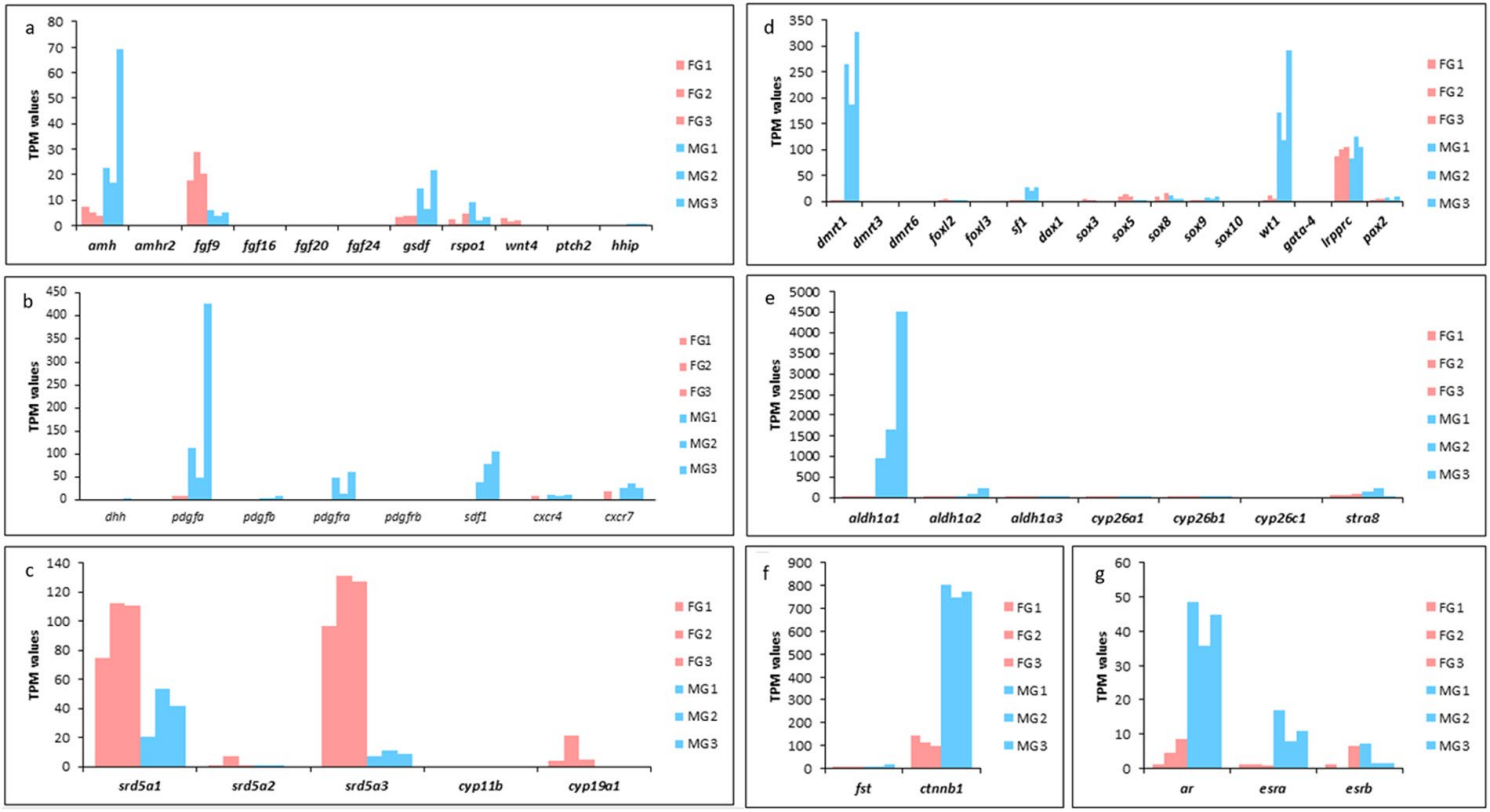
Expression levels of gametogenesis and sex-differentiation related genes in *C. orientalis* gonad transcriptomes. (**a**) Growth factor and receptor genes I. (**b**) Growth factor and receptor genes II. (**c**) Steroidogenic enzyme genes. (**d**) Transcription factor genes. (**e**) Meiosis regulation genes. (**f**) Follistatin and β-catenin. (**g**) Steroid hormone receptor genes.

Evaluation of the expression profiles in gonads revealed 14 genes that are differentially expressed between the two sexes and may play important roles in sex differentiation, gonad maintenance, and gametogenesis (Fig. 5).

The *Anti-Müllerian hormone* (*amh*) encoded protein is involved in the regression of the Müllerian ducts, the ontogenetic precursors of the female reproductive tract, in most vertebrates, and acts with its receptor Amhr2 in triggering the development of gonadal tissue^54^. Besides the function in duct regression, this gene plays a role in spermatogenesis^55,56^. Among vertebrates, expression of this gene is variable in relation to sex and time of onset. In *Xenopus laevis* the *amh* expression has been more readily detectable in mature testes than in undifferentiated gonads^57^. In *C. orientalis* the high expression of a*mh* preferentially in males (Fig. 5a) is in line with its known involvement in proliferation and differentiation of spermatogonia^55^. This observation is in agreement with our histological analyses showing the presence of spermatocytes in seminiferous tubules (Fig. 4). Moreover, the same expression pattern that we find in the Chinese fire-bellied newt has been reported for other vertebrate species such as lungfish and teleosts^53^. The lack of identification of an orthologous *amhr2* sequence in *Cynops* is likely attributable to absence of transcripts and not a gene loss event since this gene has been found in other amphibians^58,59^.

**Figure 6:**
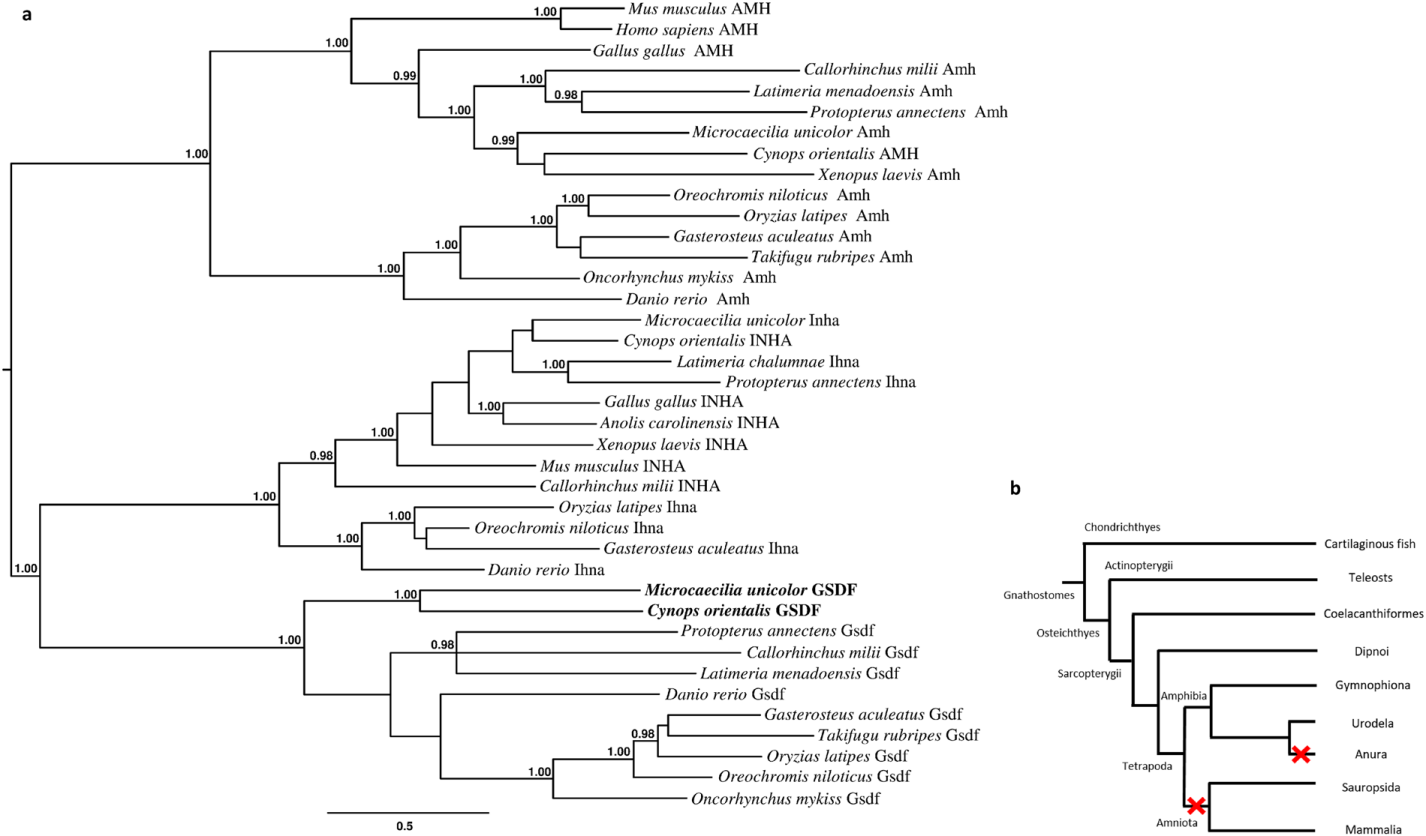
Phylogenetic attribution of GSDF amino acid sequences. (**a**) Phylogenetic tree of GSDF, AMH and Inhibin α amino acid sequences. Bayesian inference: 1,000,000 generations, sampling every 100, Jones substitution model, stationarity defined as when the average standard deviation of split frequencies approaching 0.0075, burn-in set to 2,500, midpoint rooting. In bold two amphibian GSDF sequences are highlighted. (**b**) Schematic representation of *gsdf* evolutionary history in gnathostomes. Red cross indicates gene loss.

*Gonadal Soma-Derived Factor* (*gsdf*), as *amh*, is a member of the TGF-β superfamily and is involved in testis development in fish^60^. Moreover, in some teleosts it has a role as male sex initiator^61^ or as master sex determining gene^60,62^. We have previously reported the presence of an orthologous sequence in the cartilaginous fish *Callorhinchus milii* and in the two basal sarcopterygian fish, *Latimeria menadoensis* and *Protopterus annectens*^53,63^. The analysis of *C. orientalis* transcriptome identified a bona fide *gsdf* sequence (Supplementary Fig. S3) whose orthology was confirmed by phylogenetic analysis (Fig. 6a). This finding represents the first report of a *gsdf* gene in a tetrapod species (Fig. 6b). The presence of this gene was also investigated in the genomes of *Xenopus* and *Microcaecilia unicolor*, belonging to Anura and Gymnophiona, respectively. The identification only in caecilian species (orthology confirmed by phylogenetic analysis), besides salamander, and the absence in reptiles, birds, and mammals indicate that *gsdf* was lost at least twice in the evolutionary history of tetrapods: in the Anuran lineage and in the common ancestor of Amniota. The expression analysis revealed a male biased sexually dimorphic pattern (Fig. 5a), although with values lower than those identified in basal sarcopterygian fish and teleosts^53^.

*Desert hedgehog* (*dhh*) is a member of the Hedgehog gene family, which in vertebrates includes two other homologues, *sonic hedgehog* (*shh*) and *indian hedgehog* (*ihh*)^64^. Hedgehog genes encode secreted proteins that influence the growth of several tissues during development. In humans and mice it has been showed that the lack of *dhh* expression causes infertility and testicular defects^65^. The activity of this gene persists also into the adult controlling the maintenance of male spermatogenesis and the germline^66^. Recently, among mammals also a role of DHH in ovarian development in marsupials has been proposed^67^. The expression pattern observed in *C. orientalis* (Fig. 5b) is in line with reports in another amphibian, *X. laevis*^13^, and confirms a conserved role in male gonad function.

Genes encoding members of the fibroblast growth factors (FGF) and wingless-related MMTV integration site (WNT) families direct the development of several organs. Loss of *fgf9* leads to male-to-female sex reversal^68^, while loss of *wnt4* results in partial testis development in XX mice^69^. In mouse male gonad, the sex determination gene *Sry* up-regulates *fgf9* and represses *wnt4*. These data suggested that the antagonism between Fgf9 and Wnt4 controls the sex fate of the gonad^70^. Unexpectedly, *fgf9* showed sex bias towards female in *Cynops* (Fig. 5a), like in lungfish. Similarly, *fgf9* was much higher in ovaries than testes of *Rana rugosa*. In this species, *fgf9* was expressed in early gonads of both male and female individuals, displaying no sexual dimorphism at this stage^71^. In *Xenopus laevis*, on the other hand, it showed sexual dimorphism towards male around metamorphosis^12^. Hence, a crucial role of *fgf9* for testis development in amphibians still needs to be further investigated.

Regarding the *wnt4* gene, the expected female biased expression was observed in *Cynops* (Fig. 5a). In mammals, WNT4 and RSPO1 through β-catenin stabilization (*ctnnb1* gene) lead to female gonadal fate^72^. Similar stabilization of β-catenin was proposed for *X. laevis*, where *wnt4* and *rspo1* were highly expressed in developing ovaries^12^. However, in *Cynops*, no difference in expression levels was observed for *rspo1* between ovary and testis, while a strong sex-bias towards male was observed for *ctnnb1* (Fig. 5f). The correlation between *rspo1*, *wnt4* and *ctnnb1* for ovary development seems to be not conserved in vertebrate evolution.

Members of the platelet-derived growth factor (*pdgf*) family play fundamental roles during several stages of vertebrate development^73^. In mammals, birds, and reptiles *pdgfs* and their receptors are involved in testis formation, inducing migration of cells from the mesonephros into the developing gonad^74,75^. Moreover, analysis of *pdgfa*-null mice provided evidence for the role of PDGFA and its receptor in Leydig cell development during fetal and adult stage, while *pdgfb* or *pdgfrb* knockout led to death during late gestation^76^. In *X. laevis* the upregulation of *pdgfa* and *pdgfb* genes in testis development supported involvement of the PDGF pathway in testicular organogenesis in anurans^12^. The high expression of *pdgfa* and its receptor in *Cynops* male gonads (Fig. 5b) suggests that the role of this gene in testis could be common to urodeles, not only in early developmental stages, but probably also later in gonad function.

*Stroma-cell-derived factor 1* (*sdf1*) is a member of the CXC-chemokine subfamily and is a physiological ligand for CXCR4, a member of the CXCR subfamily. SDF-1/CXCR4 chemokine signaling, is involved in proper directional migration and survival of primordial germ cells and this has been demonstrated also in *X. laevis*^14^. Moreover, recent studies have shown that SDF1 is responsible also for the postnatal maintenance of the spermatogonial stem cells in mouse testis interacting with an alternative receptor, CXCR7^77^. The higher expression of this gene compared to *cxcr4* also in *Cynops* suggests a similar function of SDF1 (Fig. 5b).

Estradiol and dihydrotestosterone (DHT) are steroid hormones that regulate vertebrate reproduction and are synthetized from testosterone. The *srd5a1-3* genes encode enzymes involved in the conversion of testosterone to DHT, the most potent androgen in tetrapods, while cytochrome P450 19A1 (*cyp19a1* or *aromatase*) converts testosterone to estradiol. These key enzymes were identified in the transcriptomes of *C. orientalis* and their expression was analyzed. *Steroid-5-Alpha-Reductase Alpha Polypeptides 1* (*srd5a1*) and *3* (*srd5a3*) are preferentially expressed in ovaries as well as *aromatase*, although at lower levels (Fig. 5c). The sexual dimorphic expression of *srd5a1* and *srd5a3* in *C. orientalis* gonads is in agreement with previous reports from lungfish^53^, frogs^78^, rat^79^, human^80,81^, and teleosts^78^. The expression in ovaries of *srd5a1* and *srd5a3* implicates the potential production of DHT whose role in amphibian gonads still need to be clarified. However, in females it is known that androgens control reproduction stimulating reproductive events in the ovary. Indeed, it has been proposed that DHT has estrogen-like properties inducing vitellogenin synthesis in hepatocytes and 17β-estradiol production in oocytes^78^.

Regarding the steroid hormone receptor genes, *androgen receptor* (*ar*) is mainly expressed in testis (Fig. 5g) indicating androgen signaling, essential for maintenance of spermatogenesis^82^. Moreover, in the Japanese frog *Rana rugosa* AR with its ligand functions has been proposed as male sex-determinant in this amphibian species^83^. The expression of *estrogen receptors* (*esrs*) in male gonads is intriguing despite the absence of expression of *aromatase* in this tissue (Fig. 5g). This could be due to a delay in expression of *esrs* compared to that of *aromatase*. The presence of *esr* transcripts in males has been observed also in *Pleurodeles waltl*^84^ and in the newt *Triturus marmoratus*^85^ where a role of estrogens in spermatogenesis has been demonstrated.

*Foxl2* (*forkhead box L2*) is a gene encoding a transcription factor, which in several species of vertebrates including amphibians has been shown to be important for ovary development and for maintaining its fate^12,86^. Studies demonstrated that FOXL2 acts on the c*yp19a1* (*aromatase*) promoter, leading to synthesis of estrogens^87^. Despite its low expression in adult gonads of *Cynops, foxl2* presented the expected female bias, confirming a conserved role of this gene (Fig. 5d).

Doublesex- and mab-3-related transcriptional factor 1 (*dmrt1*) is implicated in male sex determination and testis development in diverse metazoan phyla^88,89^. We found a strongly biased expression of *dmrt1* in *C. orientalis* male gonads in accordance with the expression profile of *dmrt1* in most other species^53,63,90^. As previously reported in basal sarcopterygians also in the newt expression of *dmrt3* was not detected (Fig. 6). In mammals, DMRT6 has a role in gametogenic programs coordinating the transition from spermatogonial development to meiosis^91^. The absence of expression of *dmrt6* gene in *Cynops* does not support a similar role in salamanders (Fig. 5d).

The *steroidogenic factor 1* (*sf1*) and the *Wilms tumor 1 gene* (*wt1*) are essential for the differentiation of adrenal glands and gonad development but also for secondary sexual characteristics^92^. The *sf1* gene shows sexually dimorphic expression with higher values in male mouse^93^, pig^94^, and in turtles^95^, while in chicken^96^ and alligators^97^ it is higher in developing female gonads. In the American bullfrog *Rana catesbeiana sf1* expression decreases with testes formation while in females it increases during ovary development^98^. In *Cynops* the expression profile of *sf1* is similar to that seen in mammals and turtles and thus opposite to that observed in frogs. Similarly, *wt1* presented a strong male bias in *Cynops* (Fig. 5d). In the frog *Rana rugosa*, however, no sexual dimorphism of *wt1* expression was observed at early stages, but a male strong sex bias was seen in adult gonads^71^. Hence, the *wt1* expression profile is similar in adult vertebrates, indicating a conserved role of this gene throughout evolution.

Transcription factors of the *SOX* (Sry-type HMG box) gene family are historically known to be important in testicular development and fertility in mammals^99^. This family contains the HMG box DNA binding domain, which is closely related to that of *Sry*, the master male sex determination gene in eutherian mammals^100^. During the sex determination period, *Sry* is expressed in the somatic gonad of XY individuals, upregulating *sox9* and leading to testis formation^101^. *Sox8* was shown to be involved in reinforcing *sox9* function and even substituting its role^102^. In *Cynops*, both *sox* genes are weakly expressed in adult gonads of both sexes, with *sox9* presenting the expected sex-bias towards males, while *sox8* shows similar expression in ovary and testis (Fig. 5d). To date, a conserved role in other vertebrate outside mammals of *sox9* as the main actor of testis development is controversial. In some teleosts it was shown to function much more downstream in the sex determination regulatory network^103^. Expression analyses from *Xenopus laevis* demonstrate that *sox9*, together with other male related genes, is upregulated in the developing testis^12^. Further studies are needed to elucidate a functional role of the *sox8* and 9 genes in amphibians.

Other members of the SOX family have been shown to be important in gonad development in vertebrates. *Sox5* is highly expressed during spermatogenesis and in fetal gonads of mice, but the exact role is still unknown^104,105^. Studies in the Japanese medaka, *Oryzias latipes*, showed that *sox5* has a role in regulating germ cell number during sex determination and disruption of this gene leads to XX female-to-male sex reversal^106^. Interestingly, different from what has been observed in most vertebrates, the expression of *sox5* in *Cynops* was higher in ovary than testis (Fig. 5d), following the pattern observed only in medaka so far^53,106^.

*Sox3* is located on the X chromosome of mammals, and the common notion is that this gene is the precursor of *Sry*. *Sox3* is highly expressed in gonads of mammals, and *sox3* ko mice have a disrupted gametogenesis with gonad dysgenesis, but no effect on sex determination was observed^107^. Notably, in *Oryzias dancena sox3* is the Y-linked male sex determination gene, pointing to the potential of this gene to be a master sex regulator^108^. In *Cynops*, no significant expression was observed in the gonad of both sexes, similar to other vertebrates, except mammals (Fig. 5d).

In reproduction gametogenesis is one of the most important processes and the morphogen retinoic acid (RA) plays a pivotal role in the regulation of meiosis in mammals^109,110^, birds^111^ and urodele amphibians^112^. The retinaldehyde dehydrogenase (ALDH1A1, ALDH1A2 and ALDH1A3) and the cytochrome P450 26 proteins (CYP26A1, CYP26B1 and CYP26C1) are the key enzymes in RA synthesis and degradation. RA upregulates the *stimulated by retinoic acid gene 8* (*stra8*) required for initiation of meiosis^113^, although *stra8* is absent in several teleost lineages^114^. In *Cynops*, no expression of *cyp26a1* and *cyp26b1* was detected, while *aldh1a1*, *aldh1a2*, and *stra8* were sexually dimorphic expressed. In particular, these genes show a higher expression in testes than ovaries (Fig. 5e). The expression of *stra8* in female gonads suggests that other enzymes are involved in RA synthesis in the ovary of *Cynops*. The low expression of *cyp26a1* is in agreement with its role as meiosis-inhibiting factor^109^. The *cyp26c1* gene has not been identified in the salamander transcriptome, however, given its presence in *Xenopus* this may be attributable to an absence of expression or a lineage specific gene loss event.

## Conclusions

Our analyses demonstrated a high level of completeness of the *Cynops orientalis* transcriptome, comparable to the in-silico transcriptomes of the fully sequenced genomes available for some amphibians. The huge genome size of *C. orientalis* estimated at 44.27 Gb represents a significant obstacle for the attainment of a complete genome assembly in the foreseeable future. Therefore, the mRNA collection obtained for the Chinese fire-bellied newt is a valid alternative molecular resource useful for gene expression studies, comparative transcriptomics and large-scale evolutionary analyses.

**Figure 7:**
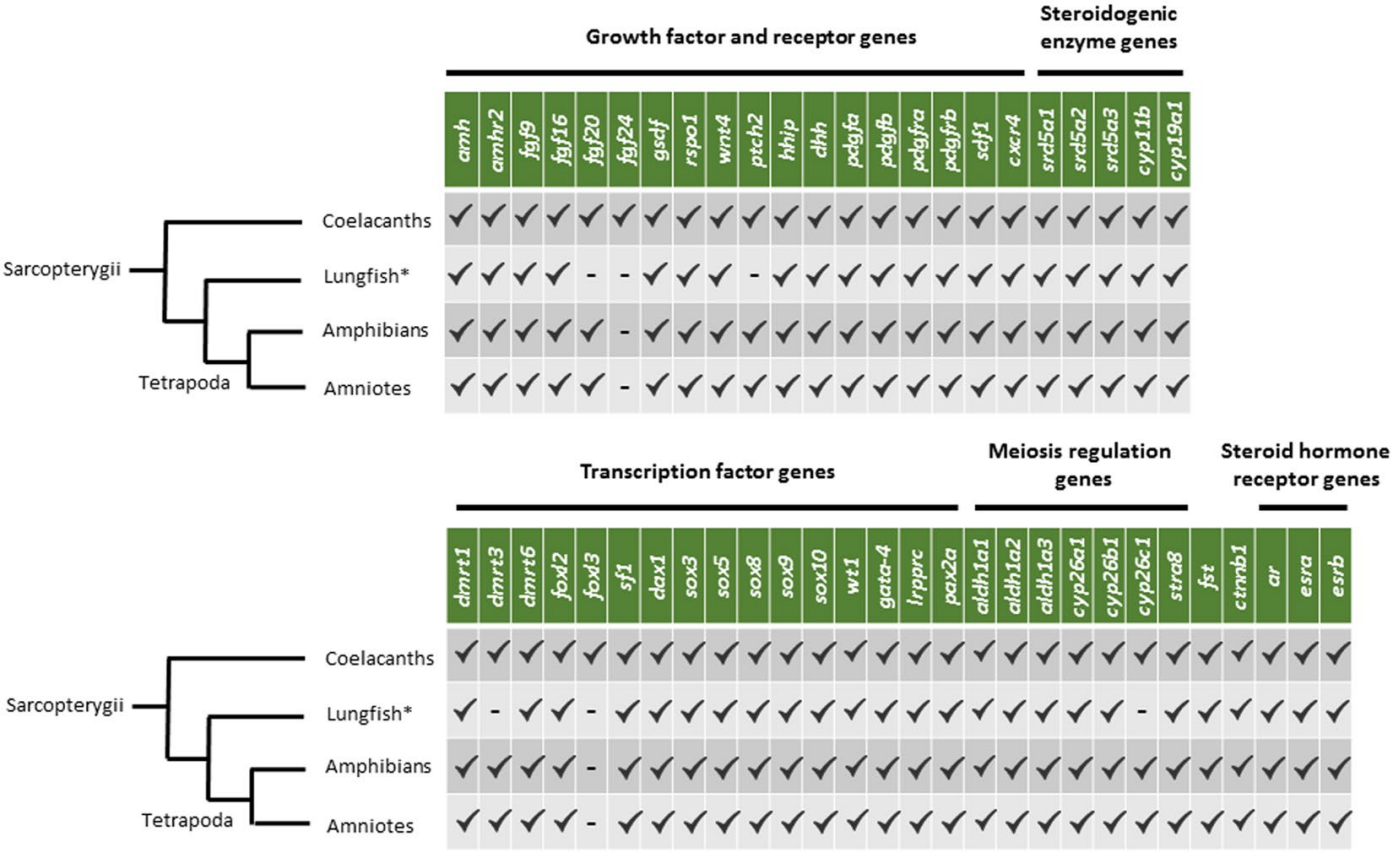
Summarizing scheme of analyzed sexual development genes in sarcopterygians. – indicates gene loss and ✓ indicates gene presence in sarcopterygians. *Indicates that data in lungfish are referred to transcriptomic resource.

The analysis of a selected gene set involved in sexual differentiation and gametogenesis performed on the transcriptomes of female and male gonads provided information on gene network evolution and function. Highly interesting is the identification of the *gsdf* gene in a tetrapod species, so far known only from bony fish and basal sarcopterygians. This finding allows to estimate the dating of the loss of this gene later in the evolution of tetrapods (Fig. 7). Our analysis did not uncover transcripts of *fgf24* and *foxl3* supporting the possible loss of both genes in the common ancestor of Rhipidistians (Fig. 7).

Although some genes, such as the growth factors *amh* and *gsdf* or the transcription factors *dmrt1* and *wt1*, showed the expected expression pattern, which suggests a conserved functional role also in *Cynops*, others displayed a unexpected diverging expression profile confirming the high variability of the sex-related pathways in vertebrates. This motivates and further in depth studies on those genes in order to elucidate the functional role in amphibians.

## Material and methods

Specimens of *C. orientalis* were obtained from a local dealer during the reproductive season. Three females and three males were anesthetized with MS222 at 2 g/l and sacrificed. All experimental procedures were approved by the Italian ethical committee Ministero della Salute (authorization n° 2E1BD.N.LYB) and all methods were performed in accordance with the relevant guidelines and regulations. Female livers, testes, and ovaries were dissected. Total RNA was extracted and used for sequencing using methodologies described in Biscotti *et al*.^31^. Pieces of the same gonads used for RNA-seq were fixed in 4% formaldehyde solution for 24 h at 4 °C. The samples were then dehydrated, embedded in paraffin, and sectioned at 5 µm thickness. The sections were counterstained with hematoxylin and eosin (HE) method.

### *De novo* transcriptome assembly and annotation

Raw paired-end reads were subjected to a trimming procedure with Trimmomatic v.0.39^115^. In detail, trimming was performed by removing Illumina sequencing adapters with the *Illuminaclip* setting, and low quality bases were detected and removed based on a sliding window approach (size = 4, minimum average quality = 15), also selecting the *leading* and *trailing* options to remove low quality read ends. Trimmed read, deposited in the NCBI SRA database under the umbrella BioProject ID PRJNA574599, were then used as an input for a *de novo* assembly with Trinity v.2.8.3, using default parameters and a minimum allowed contig length of 200 nucleotides^116^. The complexity of the assembled transcriptome was reduced with the specific aim to remove alternatively spliced isoforms and to build a non-redundant reference sequence database for gene expression studies. This was achieved with EvidentialGene (http://arthropods.eugenes.org/EvidentialGene/), recovering all the contig parts of the *okayset* and the longest isoform for each of the gene models included in the *dropset*. In addition, contigs likely to have been originated by fragmentation of longer, scarcely expressed mRNAs, contamination from exogenous sources of DNA or RNA, or by pervasive intergenic transcription, were removed following the procedure described elsewhere^31,33,117^. Contigs derived from mitochondrial DNA were detected with BLASTN^118^, based on significant similarity (e-value threshold = 1E-5) against the reference sequences (Genbank IDs: KY399474.1), and removed from the assembly.

The overall quality of the reference transcriptome assembly was evaluated with BUSCO v.3^119^. The analysis of the Benchmarking single-copy Universally Conserved Orthologs allowed the computation of completeness, duplication and fragmentation rates, with reference to the Tetrapoda OrthoDB v.9 database^120^. The Transcriptomic Diversity Index (TDI) for each sample was calculated as described in a previous publication^33^.

Functional annotation was carried out with Trinotate v.3.0.2^121^. All contigs were translated to putative protein sequences using TransDecoder v.3.0.1 and annotated based on significant BLASTX and BLASTP matches (Altschul *et al*., 1990^118^) in the UniProtKB sequence database^122^, based on an e-value threshold of 1E-5. These homologies were used to associate each contig to cell component, molecular function and biological process Gene Ontology terms^123^, to eggNOG^124^ and KEGG^125^ annotations. Protein sequences were also analyzed with Hmmer v.3.2.1^126^, searching for conserved domains included in the Pfam 31.0 database^127^.

### Identification and analysis of candidate sexual development genes

Sequences corresponding to key gametogenesis and sexual development-related genes were obtained by tBLASTN sequence homology searches performed against the *C. orientalis* transcriptomes. The retrieved transcripts were translated using Sequence Translation (https://www.ebi.ac.uk/Tools/st/) and UTRs and CDSs were identified. The orthology of each transcript was confirmed using NCBI BLAST (http://blast.ncbi.nlm.nih.gov/Blast.cgi). The sequences have been deposited in GenBank under the accession numbers (MN923216-MN923253).

Orthologous sequences used in the phylogenetic analyses were retrieved from GenBank (http://www.ncbi.nlm.nih.gov/) or ENSEMBL (http://www.ensembl.org/index.html) (for accession numbers see Supplementary Table S6). Multiple alignment of the amino acid sequences was obtained with Clustal Omega (https://www.ebi.ac.uk/Tools/msa/clustalo/) using default parameters.

Phylogenetic analyses were performed by Bayesian inference using MrBayes (version 3.2)^128^. Substitution models as defined by posterior probabilities, stationarity, generations, sampling, burnin, and specific tree building parameters and rooting details are specified in the figure legend description.

### Gene expression analysis

Trimmed reads for each of the nine biological samples analyzed were imported in the CLC Genomics Workbench v.12 environment (Qiagen, Hilden, Germany), and mapped to the non-redundant reference transcriptome. The alignment between the reads and the reference transcriptome was performed with the *RNA-seq* mapping tool, setting the length and similarity fraction parameters set to 0.75 and 0.98, respectively. Gene expression levels were computed as Transcripts Per Million (TPM)^129^, as this metric allows to efficiently compare gene expression levels both within and between samples.

The correct reconstruction of the complete open reading frame of the mRNAs of interest was checked on a case-by-case basis by visually inspecting the uniform coverage by mapped reads. Whenever necessary, the most representative (i.e. most highly expressed) isoform was recovered from the complete redundant transcriptome assembly.

Male and female gonad-specific genes were identified though a statistical analysis based on a negative binomial Generalized Linear Model (GLM)^130^, which considered in the comparison the three biological replicates for the two samples. The thresholds used for detection of significant differences between the two sexes were Fold Change > = 10 and False Discovery Rate (FDR)-corrected p-value < 1E-5.

The two sets of differentially expressed genes (DEGs) were analyzed with a hypergeometric test on annotations^131^ to identify significantly enriched GO terms or Pfam domain annotations among the male- and female-specific genes. Significant enrichment was determined for p-values lower than 0.05, paired with a difference between observed and expected values higher than 3.

TPM gene expression values were added 1 unit and log_10_-transformed before being plotted in a heat map, which clustered the transcripts showing similar expression trends based on Euclidean distance and average linkage criteria.

To enable a comparison of gene expression levels among species, we followed the strategy previously outlined in other publications^31,53,132^, recalculating gene expression levels on a subset of 1,694 unequivocal single-copy orthologs.

Transcriptomic expression data were validated by real-time quantitative PCRs performed on some candidate genes (for primer sequences see Supplementary Table S7). Reverse transcription was done using Superscript III First-strand Reaction Mix (Thermo Fisher, Invitrogen) and random primers. The reactions were performed using SYBR Green reagent and amplifications were detected with a Applied Biosystem 7900 HT. All results are averages of three independent PCR reactions from cDNA preparations of three males and three females. Transcript levels of target genes were normalized against the fire-bellied newt heterogeneous nuclear ribonucleoprotein D like (*hnrpdl)* gene (Supplementary Table S8). An additional analysis was performed using another housekeeping gene, the eukaryotic translation initiation factor 2 subunit alpha (*eif2s1*) confirming the same trend evidenced between males and females.

## Supplementary information

Supplementary information.

Supplementary information2.

Supplementary information3.

Supplementary information4.

Supplementary information5.

Supplementary information6.

Supplementary information7.

Supplementary information8.

Supplementary information9.

Supplementary information10.

Supplementary information11.

Supplementary information12.
