# Supplementary figures and images for: The transcriptome of the newt Cynops orientalis provides new insights into evolution and function of sexual gene networks in sarcopterygians
Source: Sci Rep. 2020 Mar 25;10:5445. doi: 10.1038/s41598-020-62408-x (PMC7096497; doi:10.1038/s41598-020-62408-x)

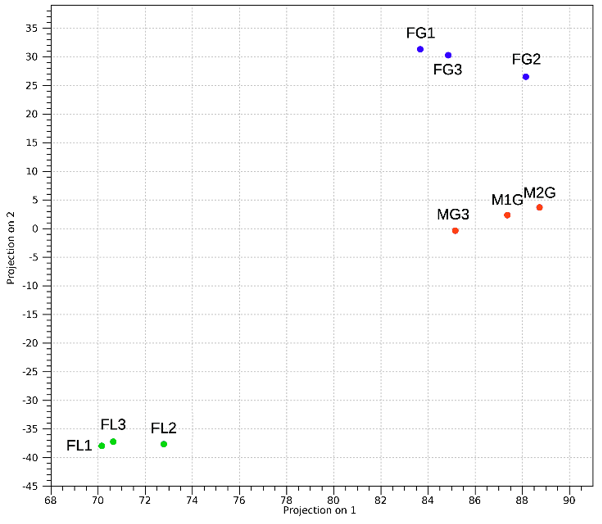

Supplement: Supplementary file 1 — Supplementary information. [file 41598_2020_62408_MOESM1_ESM.tif]

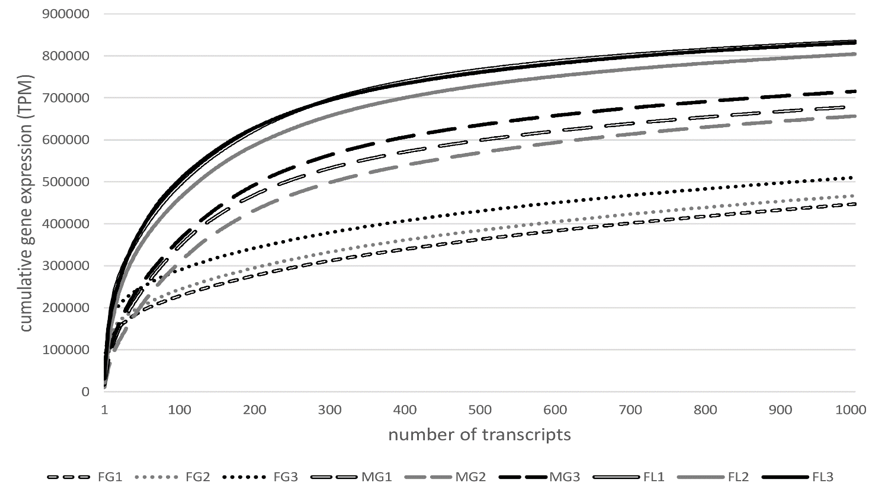

Supplement: Supplementary file 2 — Supplementary information2. [file 41598_2020_62408_MOESM2_ESM.tif]

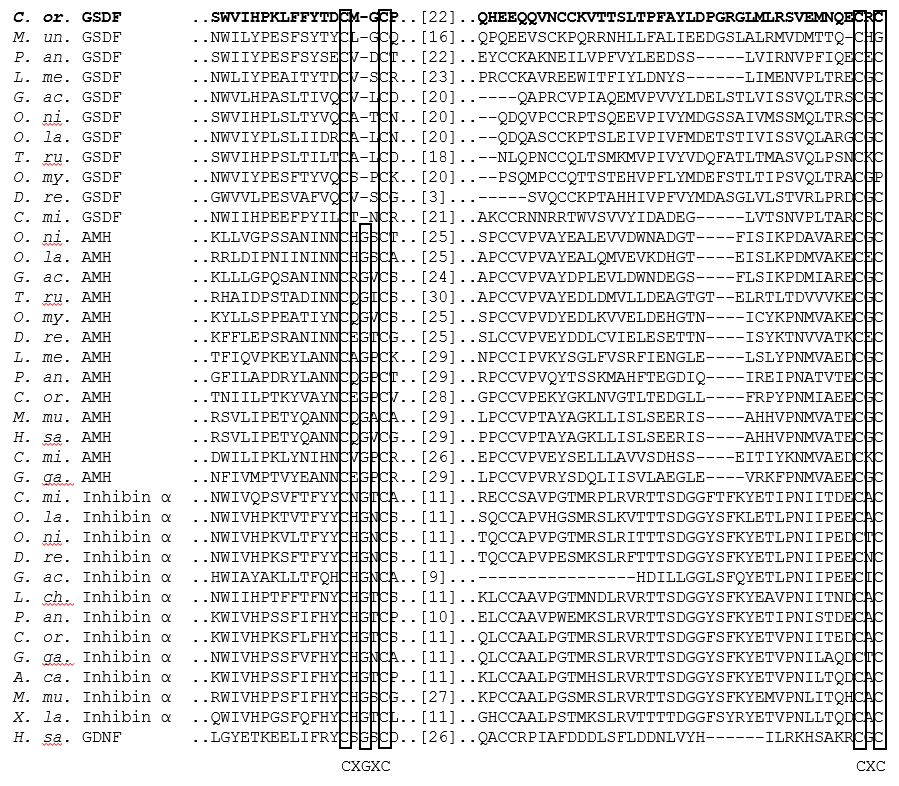

Supplement: Supplementary file 3 — Supplementary information3. [file 41598_2020_62408_MOESM3_ESM.tif]
