# Supplementary material for: The transcriptome of the newt Cynops orientalis provides new insights into evolution and function of sexual gene networks in sarcopterygians
Source: Sci Rep. 2020 Mar 25;10:5445. doi: 10.1038/s41598-020-62408-x (PMC7096497; doi:10.1038/s41598-020-62408-x)
Supplement: Supplementary file 4 — Supplementary information4. [file 41598_2020_62408_MOESM4_ESM.docx]

**The transcriptome of the newt *Cynops orientalis* provides new insights into evolution and function of sexual gene networks in sarcopterygians.**

**Maria Assunta Biscotti, Federica Carducci, Marco Barucca, Marco Gerdol, Alberto Pallavicini, Manfred Schartl, Adriana Canapa, Mateus Contar Adolfi.**

**Supplementary Fig. S1.** Principal Component Analysis of gene expression profiles of the 9 samples analyzed. FG: female gonad; MG: male gonad; FL: female liver.

**Supplementary Fig. S2.** Cumulative gene expression of the 1,000 most highly expressed genes in the nine samples.

**Supplementary Fig. S3.** Multiple alignment of GSDF, AMH and Inhibin α amino acid sequences showing the CxGxC conserved motif and the lack of the glycine residue, which is diagnostic for GSDF proteins.

**Supplementary Table S1.**Sequencing, *de novo* transcriptome assembly, and annotation summary. The three biological replicates are indicated as F1, F2, and F3 for female specimens, while were indicated as M1, M2, and M3 for male specimens. Analysed tissues were indicated as L for liver and G for gonads..

**Supplementary Table S2.** Summary report of BUSCO analysis.

**Supplementary Table S3.** Top 15 most highly expressed genes in female gonads. Gene expression levels are shown as TPM and report the average value of the three biological replicates. Transcripts lacking functional annotation have been omitted.

**Supplementary Table S4.** Top 15 most highly expressed genes in male gonads. Gene expression levels are shown as TPM and report the average value of the three biological replicates. Transcripts lacking functional annotation have been omitted.

**Supplementary Table S5.** Details of sexual development gene sequences identified in *Cynops orientalis*.

**Supplementary Table S6.** Accession numbers of sequences used in GSDF phylogenetic analysis.

**Supplementary Table S7.** Sequence of primers used for qRT-PCR.

**Supplementary Table S8.** Expression of *ar*, *fgf9* and *gsdf* by qRT-PCR in gonads of *Cynops orientalis*. The relative expression values were calculated with 2^-ΔΔCt^ method. The upper table is referred to the *hnrpdl* housekeeping while the lower table to the *eif2s1* housekeeping.
