# Supplementary material for: The transcriptome of the newt Cynops orientalis provides new insights into evolution and function of sexual gene networks in sarcopterygians
Source: Sci Rep. 2020 Mar 25;10:5445. doi: 10.1038/s41598-020-62408-x (PMC7096497; doi:10.1038/s41598-020-62408-x)
Supplement: Supplementary file 5 — Supplementary information5. [file 41598_2020_62408_MOESM5_ESM.docx]

**Supplementary table 1.** Sequencing, *de novo* transcriptome assembly, and annotation summary. The three biological replicates were indicated as F1, F2, and F3 for female specimens, while were indicated as M1, M2, and M3 for male specimens. Analysed tissues were indicated as L for liver and G for gonads.

| **sequencing output (clean reads)** | |
| --- | --- |
| F1L | 51,363,864 |
| F2L | 47,892,384 |
| F3L | 48,048,274 |
| F1G | 39,678,734 |
| F2G | 46,642,618 |
| F3G | 43,842,956 |
| M1G | 43,861,250 |
| M2G | 49,592,478 |
| M3G | 51,509,058 |
| **assembly and annotation metrics** | |
| number of contigs | 45,831 |
| mean length | 1,594 bp |
| N50 | 3,475 bp |
| total assembly size | 73,056,960 bp |
| contigs with BLASTx hits | 43.55% |
| contigs with ORFs (>100 aa) | 42.84% |
| EggNOG annotation rate | 31.20% |
| KEGG annotation rate | 38.76% |
| Gene Ontology annotation rate | 41.61% |
