# Supplementary material for: The transcriptome of the newt Cynops orientalis provides new insights into evolution and function of sexual gene networks in sarcopterygians
Source: Sci Rep. 2020 Mar 25;10:5445. doi: 10.1038/s41598-020-62408-x (PMC7096497; doi:10.1038/s41598-020-62408-x)
Supplement: Supplementary file 7 — Supplementary information7. [file 41598_2020_62408_MOESM7_ESM.docx]

**Supplementary table 3.** Top 15 most highly expressed genes in female gonads. Gene expression levels are shown as TPM and report the average value of the three biological replicates. Transcripts lacking functional annotation have been omitted.

| **contig name** | **annotation** | **TPM** |
| --- | --- | --- |
| TRINITY_DN108570_c6_g1_i1 | Histone H4 | 41,242.90 |
| TRINITY_DN105105_c2_g4_i2 | Histone H2A.J | 17,990.30 |
| TRINITY_DN103060_c3_g2_i4 | Histone H4 | 14,837.50 |
| TRINITY_DN98949_c0_g1_i3 | Histone H4 | 10,258.60 |
| TRINITY_DN102266_c2_g1_i4 | Histone H4 | 9,615.47 |
| TRINITY_DN98248_c4_g2_i1 | Histone H2A.J | 9,123.52 |
| TRINITY_DN95060_c5_g3_i1 | Histone H2A type 1 | 9,039.66 |
| TRINITY_DN108570_c2_g2_i1 | Histone H4 type VIII | 8,910.70 |
| TRINITY_DN95060_c6_g2_i1 | Histone H2B type 1-K | 8,738.63 |
| TRINITY_DN95060_c6_g1_i1 | Histone H2B 1.2 | 7,296.62 |
| TRINITY_DN100922_c4_g1_i4 | Histone H3 | 5,721.47 |
| TRINITY_DN100922_c4_g2_i1 | Histone H3.2 | 5,605.51 |
| TRINITY_DN107571_c56_g3_i1 | Regulator of rDNA transcription protein 15 | 5,590.69 |
| TRINITY_DN91637_c0_g1_i2 | Histone H1C | 3,078.19 |
| TRINITY_DN99156_c0_g1_i1 | Histone H3, embryonic | 2,373.39 |
