# Supplementary material for: The transcriptome of the newt Cynops orientalis provides new insights into evolution and function of sexual gene networks in sarcopterygians
Source: Sci Rep. 2020 Mar 25;10:5445. doi: 10.1038/s41598-020-62408-x (PMC7096497; doi:10.1038/s41598-020-62408-x)
Supplement: Supplementary file 8 — Supplementary information8. [file 41598_2020_62408_MOESM8_ESM.docx]

**Supplementary table 4.** Top 15 most highly expressed genes in male gonads. Gene expression levels are shown as TPM and report the average value of the three biological replicates. Transcripts lacking functional annotation have been omitted.

| **contig name** | **annotation** | **TPM** |
| --- | --- | --- |
| TRINITY_DN107571_c56_g3_i1 | Regulator of rDNA transcription protein 15 | 10568,87 |
| TRINITY_DN93681_c2_g2_i1 | Non-histone chromosomal protein HMG-17 | 7140,81 |
| TRINITY_DN92451_c1_g1_i1 | Ornithine decarboxylase antizyme 3 | 5530,56 |
| TRINITY_DN107571_c56_g2_i2 | Protein NLRC3 | 5418,66 |
| TRINITY_DN91514_c2_g3_i3 | Hemoglobin subunit alpha-1 | 4836,93 |
| TRINITY_DN101032_c3_g2_i1 | Hemoglobin subunit beta-1 | 4443,81 |
| TRINITY_DN88014_c3_g1_i4 | Actin, cytoplasmic 1 | 4318,06 |
| TRINITY_DN101271_c2_g1_i2 | Ferritin heavy chain B | 4265,75 |
| TRINITY_DN108252_c4_g2_i1 | Actin, cytoplasmic 1 | 4222,33 |
| TRINITY_DN94041_c0_g2_i4 | 60S ribosomal protein L36a | 3980,96 |
| TRINITY_DN108252_c4_g1_i3 | Actin, muscle | 3771,79 |
| TRINITY_DN101271_c2_g3_i1 | Ferritin | 3358,36 |
| TRINITY_DN95102_c5_g1_i3 | 40S ribosomal protein S20 | 3138,53 |
| TRINITY_DN101040_c1_g1_i9 | 60S ribosomal protein L37 | 2985,76 |
| TRINITY_DN93197_c5_g2_i3 | Elongation factor 1-alpha | 2961,59 |
